# Evolution particulière de variants cytogénétiques complexes de leucémies myéloïdes chroniques traitées par l'Imatinib

**DOI:** 10.11604/pamj.2013.15.132.2207

**Published:** 2013-08-11

**Authors:** Salifo Sawadogo, Francis Michel Hien, Rasmané Béogo, Aboubacar Toguyeni, Georges Anicet Ouédraogo

**Affiliations:** 1 Laboratoire d'hématologie et d'immunologie Centre Hospitalier Universitaire Sourô Sanou 01 BP 676 Bobo Dioulasso 01, Burkina Faso; 2 Médecine interne Centre Hospitalier Universitaire Sourô Sanou 01 BP 676 Bobo Dioulasso 01, Burkina Faso; 3 Stomatologie Centre Hospitalier Universitaire Sourô Sanou 01 BP 676 Bobo Dioulasso 01, Burkina Faso; 4 Centre International de Recherche pour le Développement de l'Elevage en zone Subhumide 01BP 454 Bobo Dioulasso 01, Burkina Faso; 5 Laboratoire de Recherche en Sciences de la Santé et en Biotechnologie Animale, Institut du Développement Rural, Université Polytechnique de Bobo Dioulasso 01BP 1091 Bobo Dioulasso 01, Burkina Faso

**Keywords:** Leucémie myéloïde chronique, complexe variant, évolution cytogénétique, Chronic myeloid leukemia, complex variant, cytogenetic evolution

## Abstract

Les variants cytogénétiques simples et complexes constituent 5 à 10% de tous les cas de leucémie myéloïde chronique. Le mécanisme de leur formation a été proposé par certains auteurs. Les aspects clinique, thérapeutique et pronostique ne sont pas différents des formes classiques à l'aire des anti-tyrosines kinases. Nous rapportons deux cas traités par Imatinib dont l'évolution cytogénétique a été particulière. Les deux patients ont été inclus dans le programme GIPAP après signature d'un consentement éclairé. Chaque patient a bénéficié d'un examen clinique, d'un hémogramme, d'un myélogramme, d'un caryotype et ou d'une hybridation intra-génique avec fluorescence avant inclusion dans le programme. Chaque patient après inclusion a été traité avec l'Imatinib à la dose quotidienne de 400mg. La surveillance clinique, hématologique et cytogénétique et moléculaire a été faite selon les recommandations de LeukemiaNet.

## Introduction

La leucémie myéloïde chronique est une néoplasie myéloproliférative chronique caractérisée classiquement par une translocation réciproque entre les chromosomes 9 et 22. L'anomalie débute au niveau de la cellule souche totipotente et la prolifération est prédominante sur la lignée granuleuse. Les cellules issues de cette prolifération clonale sont sans apoptose et sont non adhérentes. Des variants simples impliquant soit le chromosome 9 et un autre chromosome autre que le 22 ou impliquant le chromosome 22 et un autre chromosome autre que le 9 ont été décrits [1, 2]. Des variants cytogénétiques complexes impliquant les chromosomes 9,22 avec 1,2 ou 3 autres chromosomes sauf le Y ont été aussi décrits [3–5]. Nous rapportons deux cas de variants dont l'évolution a été particulière: le premier, de complexe est devenu une forme classique et le deuxième de variant simple est devenu un variant complexe tout en restant en phase chronique. L'objectif de ce travail est de décrire les particularités évolutives de variants cytogénétiques de leucémie myéloïde chronique

## Patients et observations

Les patients, deux hommes ont présenté des signes clinique et hématologique de leucémie myéloïde chronique mis en évidence suite à un examen clinique, un hémogramme et un myélogramme. Les patients ont été inclus dans le programme “Glivec international patients assistance program” (GIPAP). La surveillance a été selon les recommandations de European Leukemia Net [6].

Critères d'inclusion: présenter des signes clinique et hématologique d'une leucémie myéloïde chronique, confirmés par la mise en évidence du chromosome Philadelphie ou du gène BCR-ABL ou du transcrit BCR-ABL; signer un consentement éclairé; etre capable d'assurer les examens de surveillance.

### Cas 1

Un jeune homme de 30 ans qui nous a été adressé par la chirurgie pour hépatomégalie, splénomégalie (débord 13cm) et hyperleucocytose chez un patient qui a été opéré pour péritonite purulente par perforation appendiculaire. L'hémogramme de départ: GB:143000/mm^3^ avec une discrète myélémie, anémie normochrome normocytaire (6,2g/dl), Plaquettes:439000/mm^3^. Le myélogramme de grade 4+ avec une lignée granuleuse à 87% à prédominance myélocytaire. Le caryotype a mis en évidence:46, XY, une t (9;11;22) (q34;q24;q11) sur 100% des mitoses examinées. Les examens de biochimie usuelle ont été normaux. Mis sous Imatinib à raison 400mg/j en prise unique, en deux mois une rémission hématologique complète a été obtenue, normalisation du caryotype en 9 mois de traitement mais la FISH a montré 6% de BCR-ABL. Le contrôle du caryotype après deux mois a trouvé: 46, XY, t (9;22) (q34;q11) dans 100% des mitoses. Quatre mois après on a obtenu une rémission cytogénétique complète et la recherche du transcrit a montré un ratio BCR-ABL/ABL à 02% avec un seuil de détection à 0,01. En raison d'une thrombopénie menaçante, le traitement a été interrompu. Le patient devait revenir régulièrement pour une surveillance et reprendre le traitement si la situation s'améliorait. Après trois mois de surveillance, il a disparu pendant 19 mois et revenu en rechute en phase blastique. Il est décédé sans que nous puissions réaliser les examens de génétique et cytogénétique.

### Cas 2

C'est un jeune homme de vingt ans venu en consultation pour: amaigrissement non chiffré, pâleur conjonctivale, asthénie, splénomégalie débordant de vingt quatre centimètres le grill costal (162mm à l'échographie) hépatomégalie débordant de huit centimètres le grill costal (153mm de flèche hépatique à l'échographie). L'hémogramme de départ est le suivant: Globules blancs:810300/mm3 avec une importante myélémie à prédominance myélocytaire, une anémie macrocytaire normochrome et les plaquettes à 423000/mm^3^. Le myélogramme a trouvé un os dur, une moelle très riche, avec une lignée granuleuse à 89% prédominance polynucléaire neutrophile. Il a été mis sous Hydroxyuré 2g/j en deux prises et un mois plus tard un caryotype et une hybridation in situ avec fluorescence ont été demandés. Les résultats ont été les suivants:46, XY, t (11;22) (q23;q12) et absence de réarrangement BCR-ABL sur les mitoses et sur les cellules interphasiques. Un topogramme a été demandé pour rechercher un éventuel sarcome d'Ewing, cet examen a été normal. Nous avons quand même débuté un traitement avec l'Imatinib à la dose de 400mg/j en une prise. Le succès avec cette molécule nous a amené à demander la recherche du transcrit BCR-ABL qui s'est avéré positive avec un réarrangement M BCR-ABL de type b2-a2. Un second caryotype a été demandé cinq mois après le premier et il a révélé un variant complexe: 46, XY, t (9;11;22) (q34;q23;q11) sur 30% des mitoses examinées. La rémission cytogénétique complète a été obtenue après 40 mois de traitement avec l'Imatinib chez un patient particulièrement indiscipliné et observant mal son traitement.

## Discussion

Des auteurs ont émis l'hypothèse des mécanismes de formation des variants complexes de LMC [2, 3]. La première hypothèse serait que les cassures seraient simultanées au niveau des chromosomes suivies de translocation circulaire dans une cellule souche. La deuxième hypothèse serait qu'il y aurait une translocation classique dans une ou plusieurs cellules souches suivie d'une ou de plusieurs autres translocations additives dans des cellules progénitrices précoces ou tardives. Autrement dit ces anomalies cytogénétiques additives traduiraient la progression de la maladie. D'autres mécanismes ne seraient pas à écarter.

Notre premier cas serait en adéquation avec la deuxième hypothèse. Le passage de la forme complexe à la forme classique s'est déroulé en période de rémission hématologique complète. Le clone classique donc serait donc apparu que dans les cellules souches avec des anomalies secondaires additives dans les cellules progénitrices.

Des auteurs comme Ishihara T et al [7], ont affirmé que la translocation ne peut survenir que si la cassure se faisait en 9q34 et en 22q11. C'est peut être ceci qui explique que lors du premier caryotype du deuxième patient, on n'a pas pu mettre en évidence le chromosome Philadelphie. Le passage de la forme variante simple à la forme de variant complexe s'est déroulé durant la phase chronique de la maladie. Il se peut qu'il y ait eu une insertion équilibrée entre le 9 et le 22 et non une translocation. Cela expliquerait la négativité de l'hybridation intra génique en fluorescence. Cette insertion a été probablement suivie d'une cassure secondaire puis d'une translocation complexe dans le même clone cellulaire. Les contrôles du caryotype après la rémission hématologique jusqu'à la rémission cytogénétique complète n'ont pas mis en évidence un changement dans la forme cytogénétique. A moins qu'entre l'avant dernier contrôle et le dernier contrôle, un changement se soit produit, échappant à notre surveillance. S'il n'y a pas eu de changement, le cas de ce patient confirmerait la première hypothèse émise par les auteurs ci-dessus. Qu'il y ait eu ou non un changement de forme, comment expliquer que de forme cytogénétique classique elle devienne complexe puis variant simple? Ou bien de variant cytogénétique complexe, elle devienne un variant cytogénétique simple. À moins que comme a dit Huret J.L. et al [2] le simple variant soit en réalité un variant complexe impliquant quatre chromosomes. Tout cela traduit une instabilité chromosomique dont le(s) facteur(s) déclenchant(s) nous serait (seraient) inconnu(s). En effet le seul facteur connu responsable de la survenue des LMC est les radiations ionisantes. On a longtemps incriminé le Benzène mais des études de méta-analyse ont exclus ce facteur [8]. Des études de grandes séries menées en Amérique du Nord, en Europe, en Asie, en Australie et Afrique du sud ont trouvé une prévalence élevée des variants de leucémie myéloïde chronique chez les noirs en Afrique du sud et aux états unis. Des facteurs ethniques et environnementaux ont été incriminés pour expliquer ces prévalences élevées et donc l'instabilité chromosomique [9].

## Conclusion

La survenue des variants cytogénétiques complexes est probablement liée à des facteurs ethniques et environnementaux. Leur évolution cytogénétique sous Imatinib est probablement fonction de leur mécanisme d'installation. Le pronostic de ces variants à l'aire des anti-tyrosines Kinases n'est différent des formes classiques.
